# A Multimodal Amphibious Robot Driven by Soft Electrohydraulic Flippers

**DOI:** 10.34133/cbsystems.0253

**Published:** 2025-06-09

**Authors:** Fuyi Fang, Junfeng Zhou, Yuanzhen Zhang, Yanran Yi, Zhen Huang, Yicheng Feng, Kai Tao, Wenbo Li, Wenming Zhang

**Affiliations:** ^1^ State Key Laboratory of Mechanical System and Vibration, School of Mechanical Engineering, Shanghai Jiao Tong University, Shanghai 200240, China.; ^2^ School of Aerospace Engineering and Applied Mechanics, Tongji University, Shanghai 200092, China.; ^3^ The Ministry of Education Key Laboratory of Micro and Nano Systems for Aerospace, School of Mechanical Engineering, Northwestern Polytechnical University, Xi’an710072, China.

## Abstract

Soft robots are exceptionally suited to exploring complex environments, including amphibious navigations, due to their flexible and adaptive characteristics. However, achieving efficient actuation and multimodal locomotion or transition in amphibious environments for soft robots is challenging. In this paper, we present a multimodal amphibious robot with radial symmetry configuration and 3 different locomotion modes (crawling on land and underwater, swimming in water). The robot consists of 3 soft electrohydraulic flippers, which can be independently or synergistically actuated to rotate or oscillate in both air and water and generate the propulsion for amphibious locomotion. Theoretical analysis and experimental tests have verified the remarkable amphibious actuation performance of the soft electrohydraulic flippers with effective electrode encapsulation process. Optimal actuation frequencies are also obtained for maximizing the efficiency of the robot’s movements in different mediums. Based on the 3 powerful soft electrohydraulic flippers with radial symmetry distribution, the robot can smoothly transition from crawling on land to crawling underwater, and swim up from the bottom to the surface of water, without reconfiguration of the robot. This work demonstrates the first amphibious soft robot based on electrohydraulic actuators with multimodal locomotion transition in an amphibious environment and may open up more possibilities for the development of multimodal soft robots.

## Introduction

The capacity of soft robots to navigate intricate, unstructured environments has been acknowledged [1–3], largely due to their compliant bodies and high degree of freedom. From the minutiae of the human intestine [4–6] to the challenging topographies of the natural world [7–9], soft robots have demonstrated considerable potential for exploration. In such applications, it is inevitable that the robot will encounter situations that change the environmental medium. For example, when the robots enter water from land [10–12] or float up to surfaces from water [13,14], they will exhibit different actuation characteristics in aquatic and non-aquatic environments, as well as the different contact conditions with solid surfaces, which can result in altered or even degraded performance. Therefore, the question of how to achieve efficient actuation of soft robots in multiple media is such an interesting and meaningful topic.

At present, a number of different approaches have been taken to the actuating of soft robots, including the use of pneumatics [15,16], shape memory alloys [17,18], magnetic matters [19,20], and other smart materials, such as light-sensitive [21,22], heat-sensitive [23–25], and humidity-sensitive [26,27] materials. In environments characterized by instability, the use of smart materials to drive soft robots presents inherent challenges, as the ambient temperature, humidity, light conditions, and even media are all uncertain factors that may change over time, which makes it difficult to control the behavior of smart materials [28]. In comparison, pneumatic, shape memory alloy, and magnetic actuations are more robust and facilitate a greater range of motion modes due to their highly designable structures. To illustrate, pneumatic actuators are capable of rolling, crawling, and jumping on land [29,30], as well as swimming and paddling in water [31,32]. The use of buoyancy enables the pneumatic actuator to achieve rapid uplift in the water, thereby facilitating highly maneuverable movement. However, the buoyancy effect also results in instability in the horizontal position of the robot in the water while inflating and deflating, which is typically an undesired outcome. The use of shape memory alloys has been employed to adapt the local configuration of soft robots [33] in order to facilitate their ability to deal with changes in the surrounding environment. However, such approaches are constrained by the efficiency of energy utilization and the frequency of actuation. Magnetic-actuated soft robots represent an optimal solution for the inspection of small-scale environments. They are designed to be sufficiently compact to achieve crawling [6,34], tumbling [35,36], and other motions [37,38] in the presence of a magnetic field. Furthermore, the magnetic field can be readily adjusted, enabling the soft robots to cope with a vast range of media and contact surfaces. Nevertheless, the apparatus necessary to generate the magnetic field markedly constrains the deployment of magnetic-actuated robots on larger scales, and exploring in open natural environments is clearly unsuitable.

The use of electrostatic actuation allows for the creation of an efficient and stable actuator for use in multimodal amphibious soft robots. Typical electrostatic actuators include dielectric elastomers [39–41], which are designed to stretch and bend by squeezing an elastic membrane with the attraction of electrodes. These actuators have relatively low energy consumption because the actuation process is similar to the charging and discharging of a capacitor. By encapsulating the electrodes, they are also allowed to function in aqueous environments [42–44], enabling the intricately designed robots to swim. Some robots based on dielectric elastomers also realize the crossing between different mediums. They use multilayer sandwich actuator [45,46] or cylindrical linear actuator [47] to make the robot capable of moving on land as well in water, which ensures the rapid switching of exploring environments. In order to deal with the interference caused by the switching from external factors like the surface tension of the liquid and the viscosity coefficient of different mediums, there are 2 common methods: the first is to change the drive frequency [47], as dielectric elastomers show optimal frequencies in different mediums; another method is to change the movement mode [46], for example, when entering the water, rowing or swimming instead of crawling tends to have better efficiency. In recent years, another type of electrostatic actuator, known as the hydraulically amplified self-healing electrostatic (HASEL) actuator [48,49], has attracted considerable interest. This alternative design replaces the solid dielectric of the dielectric elastomer with liquid dielectric, thereby converting the squeezing of the electrodes into hydraulic pressure [50,51]. In comparison to dielectric elastomers, electrohydraulic actuators exhibit a greater driving force and amplitude of motion, rendering them more suitable for use as thrusters [52]. A variety of configurations have been developed for existing electrohydraulic actuators, enabling them to achieve tumbling, jumping, and crawling on land [53,54], as well as fluttering [55] and swimming [56,57] in water. These robots have demonstrated the ability to perform efficient motions in different medium. However, the transformation of these modes requires adjustments to the overall structure of the robot, which creates discontinuities in the motions. In light of the capabilities of amphibians, we consider optimizing the structure of the electrohydraulic actuator so that it can seamlessly traverse different medium without altering its overall configuration. This approach aims to enable locomotion on land and in water in multiple modes.

**Fig. 1.**
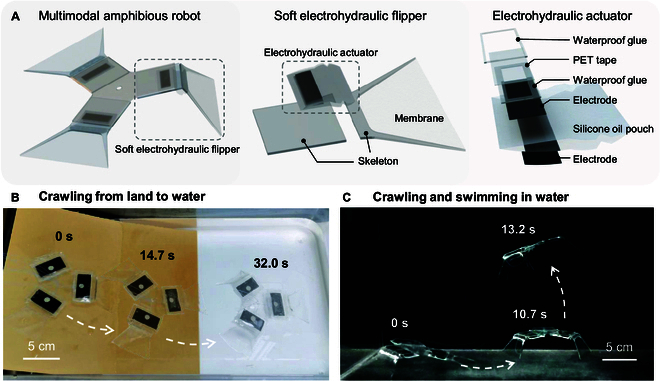
The multimodal amphibious robot driven by electrohydraulic actuators. (A) Construction of the amphibious robot, the soft electrohydraulic flipper, and the encapsulated electrohydraulic actuator. (B) Process of the robot crawling from land to water. (C) Process of the robot crawling underwater and swimming in water.

The objective of this proposal is to develop a multimodal amphibious robot driven by soft electrohydraulic flippers, with a basic bend-type actuator to enable the robot to crawl from land to water, as well as swim in water, which are illustrated in Fig. 1. In contrast to previous electrohydraulic-actuated robots, this design not only enables the robot to operate on land efficiently but also adapts it to underwater environments. The different working mechanisms of the actuator on land and in water are analyzed by adopting the method of single-sided electrode encapsulation, which realized the amphibious movement of the actuator in principle. Furthermore, this work explored a multimode movement mechanism and designed a corresponding unique flipper structure, taking into account the distinct environmental characteristics of both mediums, and allowing the robot to generate sufficient friction on land while simultaneously achieving a substantial propulsion force in water. This enables the robot to crawl on land, paddle underwater, and swim in water while also facilitating a seamless transition between the 2 mediums and multiple movement modes. A force–electro-hydraulic coupling model of the actuator is developed to reveal the effect of voltage on the bending angle of the actuator, and the output moment of the actuator is characterized experimentally as well. The symmetric triple actuator design enables the robot to move omnidirectionally in a plane, and to enhance movement efficiency, the input signals and actuation frequency are optimized to achieve a maximum movement speed of 2.9 cm/s on land. In water, driving the robot with different numbers of flippers enables the robot to switch between crawling underwater and swimming in the water. The motion mechanism of the robot in water has been subjected to detailed investigation, with particular focus on the simulation and analysis of the complex process of swimming. This has enabled the change of flow field during swimming to be revealed. It is noteworthy that the amphibious robot exhibits excellent temperature adaptability, with experiments indicating that it can operate within a temperature range of 2.1 to 61.3 °C. This further enhances its environmental adaptability. The robot is capable of crawling from land into water smoothly and subsequently swimming from underwater to the surface without undergoing any structural transitions. This is the first instance of an electrohydraulic actuator being utilized continuously in an amphibious environment, thereby addressing a previously identified limitation in the field of cross medium actuation by these actuators.

## Materials and Methods

### Design of the multimodal amphibious robot

The proposed multimodal amphibious robot consists of 2 principal components: the body skeleton and the soft electrohydraulic flippers, as shown in Fig. 1A. There are 3 flippers distributed on the body skeleton in a circumferential symmetrical manner with an angle of 120° to each other. The 3 flippers are capable of moving the robot in specific directions, which are referred to as 3 basis vectors. These basis vectors form a 120° angle with each other and can be combined to form any vector on the plane, thereby enabling the robot to reach any location on the plane. It should be noted that the single flipper is only capable of moving the robot in a single direction. Consequently, the coefficients of each direction vector can only be positive. This is the reason why at least 3 actuators (rather than 2) are required to achieve omnidirectional movement on the plane.

In order to enable the robot to move on land and in water, as well as to satisfy the need for high-frequency motion, electrohydraulic actuators are selected as the flippers of the amphibious robot. A pair of skeletons is added to one side of the electrohydraulic actuator in order to restrict the expansion of the fluid pouch. We articulate the 2 parts of the skeleton in the middle; therefore, the contraction motion of the electrohydraulic actuator is limited to becoming a bending motion. The skeleton is constructed from a 1-mm-thick acrylic sheet by laser cutting, and its configuration has been meticulously devised to align with the disparate requirements of movement on land and in water. The front section of the skeleton has been designed in the shape of a flipper and is covered with a thermoplastic urethane (TPU) film in order to adapt to the paddling motion in an aquatic environment and to increase the propulsive force. The rigid frame of the flipper tapers to a pinpoint shape in order to increase friction during contact with the terrain surface, thus improving movement efficiency.

In addition to the design of the skeleton, the existing electrohydraulic actuator has been enhanced to ensure its adaptability to both land and water environments, thereby guaranteeing its reliability. Previous electrohydraulic actuators designed for use in non-aqueous environments comprise a liquid pouch filled with silicone oil and electrodes on both sides [58]. In this work, the pouch is an approximate rectangle measuring 30 mm × 40 mm, with a curved protrusion at the end of the 30-mm side, as illustrated in Fig. S1, which is designed for enhancing the bending performance [55,56]. The irregular shape of the pouch is created by sealing two 20-μm polyethylene terephthalate–ethylene vinyl acetate (PET-EVA) composite films under the heat of a fused deposition modeling (FDM) 3D printer. Additionally, 2 polyimide (PI) films are laid on the outside of the 2 layers of PET-EVA composite films during the printing process, which ensures the flatness of the 2 intermediate layers (as seen in Fig. S3A). Subsequently, electrodes are printed on both sides of the pouch via a screen-printing process. The electrode material is carbon paste, and the process platform is illustrated in Fig. S3B. The quantity of the injected silicone oil is 1.2 ml, which is fit to the size of the designed pouch. This allows the silicone oil to exactly fill the curved protruding area of the pouch, thereby optimizing the bending performance. In order for the actuator to function effectively in underwater environment, it is essential to ensure that one side of the electrode is adequately waterproofed. To this end, we have developed a multilayer waterproofing process, comprising the following steps: First, a layer of waterproof soft adhesive is applied around the electrode; then, a 60-μm-thick PET tape is placed over the adhesive; subsequently, another layer of waterproof soft adhesive is applied around the tape to fill any gaps; and finally, the adhesive is cured with ultraviolet (365-nm wavelength) light. The 3-layer process has demonstrated effective waterproof performance in the subsequent experiments and validations.

### Working principle of the soft electrohydraulic flipper

**Fig. 2.**
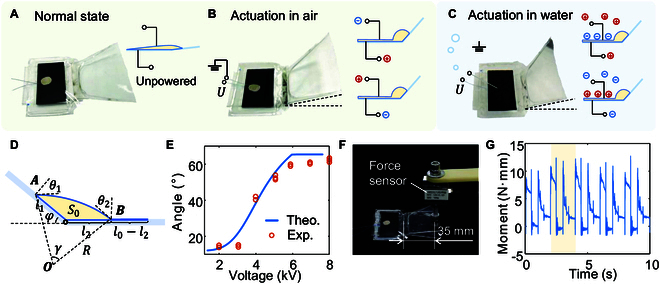
Design of the soft electrohydraulic flipper. (A) Normal state of the soft electrohydraulic flipper. (B) Working principle of the actuated soft electrohydraulic flipper in air. (C) Working principle of the actuated soft electrohydraulic flipper in water. (D) Modeling of the soft electrohydraulic flipper. (E) Theoretical and experimental result of the flipper’s bending angle, considering different input voltage. (F) Experiment platform for moment measuring. (G) Measured moment of the flipper with the frequency of 1 Hz and input voltage of 6 kV.

The electrohydraulic actuator is capable of functioning in both terrestrial and aquatic environments, exhibiting distinct operational characteristics in each context. As illustrated in Fig. 2A, in the absence of an external power source, the dielectric liquid within the actuator occupies the entirety of the pouch. Consequently, the pressure within the dielectric liquid is relatively low, resulting in a negligible tension within the pouch membrane and a correspondingly minimal pulling force on the skeleton. This ultimately leads to the actuator exhibiting minimal bending angles. In a terrestrial context, the electrodes on either side of the electrohydraulic actuator are connected to the signal source and the ground, as illustrated in Fig. 2B. Upon the application of a signal, positive and negative charges are generated on the electrodes on each side alternatively, resulting in an attractive force between the electrodes and a subsequent squeezing action on the dielectric liquid inside. Given that the electrodes situated at the distal end of the actuator are positioned in closer proximity to one another, these parts are initially attracted together, resulting in the squeezing of the dielectric liquid toward the region of the actuator that is not covered with electrodes. When the voltage is sufficiently elevated, the electrodes on both sides are fully attracted, exerting a compressive force on the dielectric liquid to form an arch column. At this juncture, the pressure within the dielectric liquid markedly intensifies, prompting a concomitant increase in the tension within the pouch film. This, in turn, exerts a force on the skeleton, causing it to assume an angle of rotation at the articulation. Upon the disconnection of the power supply, the charge on the electrodes is gradually released, resulting in their separation from one another. This, in turn, causes the actuator to revert to its original state.

The actuator functions in a manner that differs when submerged in water as opposed to on land. Given that water can generate polar particles under the influence of an electric field, it is an optimal choice to utilize water as the electrode on one side. As illustrated in Fig. 2C, the electrode on the waterproofed side is extended to connect the power supply, while the electrode on the opposing side is directly exposed to the water as the ground polar (the wire lead on the exposed electrode side can be left assembled to facilitate the conversion between land and water). Upon the application of a voltage signal, the electrode that is connected to the power supply first generates charges, which then attract opposing charges present in the water on the opposite side of the electrode. In this case, the water assumes the role of the ground pole, providing the actuator with polar particles. The electrodes on both sides are attracted to each other, resulting in an increase in pressure on the dielectric liquid within, which eventually causes the actuator to bend. Besides, with this working principle, the ionic strength of the liquid has no marked effect on the performance of the actuator, as the attracting force of the electrodes is directly proportional to the number of particles, but is unconcern with the type of particles like ionic or polar molecule. It is demonstrated in Fig. S7 that the actuators perform similarly in the tape water and salt water.

Although the principle of actuation is different, the bending effect produced is similar. Furthermore, this method enables a quick changeover between land and water actuation modes, requiring only a changeover of the wire connection.

### Modeling of the actuator

In order to gain further insight into the actuating performance of the electrohydraulic actuator and to intuitively ascertain the effect of voltage on the bending angle of the actuator, we employ an analytical method to construct a force–electric–hydraulic coupling model of the electrohydraulic actuator. The geometrical model and parameters of the cross-section of the actuator are illustrated in Fig. 2D. The length of the pouch affixed to the front skeleton is designated as $l_{1}$, while the length affixed to the back is $l_{0}$, and the length of the electrodes that contact with another is $l_{0}-l_{2}$. Additionally, the cross-sectional area of the internal dielectric liquid is represented by $S_{0}$. It is assumed that the 2 parts of the skeleton are ideally articulated with an angle of φ. The bulging portion of the pouch is represented by a circular arc with radius R, the angle of the circumcenter γ, and the angle between the circular arc and the 2 parts of the skeleton $\theta_{1}$ and $\theta_{2}$. The tension inside the film is a position-independent quantity, designated as $T_{1}$, while the pressure of the dielectric liquid inside is represented as p. All these parameters are listed in Table S1.

The modeling is divided into 3 parts, commencing with the coupling of the liquid to the solid. It is assumed that the stretching of the film is negligible, which means that the length of the film is constant, thus enabling the conservation equation to be obtained

$$l_{0}-l_{2}+\mathrm{R\gamma}=s_{0}$$ (1)

In order to determine the total length of the film, $s_{0}$, it is necessary to apply the geometrical relation $\begin{array}{c} \;R= \end{array}$ $\begin{array}{c} \frac{\sqrt{l_{1}^{2}+l_{2}^{2}+2l_{1}l_{2}\text{cos}\varphi }}{2\text{sin}\left(\gamma /2\right)},\;\gamma =\pi -\theta_{1}-\theta_{2}-\varphi \end{array}$.

As for the dielectric liquid, which is regarded as incompressible, the conservation equation can be derived

$$\frac{\gamma }{2\pi }\pi R^{2}-\frac{\text{sin}\gamma }{2}R^{2}+\frac{1}{2}l_{1}l_{2}\text{sin}\varphi =S_{0}$$ (2)

The third conservation equation introduces the variables $\theta_{1}$ and $\theta_{2}$ and describes the relationship between these 2 angles and the lengths $l_{1}$ and $l_{2}$

$$\frac{\text{sin}\left(\frac{\pi }{2}-\theta_{1}-\frac{\gamma }{2}\right)}{l_{1}}-\frac{\text{sin}\left(\frac{\pi }{2}-\theta_{2}-\frac{\gamma }{2}\right)}{l_{2}}=0$$ (3)

The second stage of the model construction process is to determine the 2 mechanical quantities, $T_{1}$ and p, which necessitates the formulation of balance equations. The initial equation is the force balance for the upper surface film of the pouch

$$\mathrm{pR}=T_{1}$$ (4)

The second balance equation describes the effect of the tension in the film and the pressure exerted by the internal liquid on the front part of the skeleton. This equation represents the balance of moments in the flippers about the articulation point, where $M_{1}$ is the equivalent moment of the load

$$pl_{1}\frac{l_{1}}{l_{2}}+M_{1}=T_{1}l_{1}\text{cos}\theta_{1}$$ (5)

It is finally demonstrated that the force is coupled to the electrostatic force. For the portion of the upper and lower electrodes that have been attached, it is assumed that a thin dielectric liquid film is still present between them and will provide the pressure p. The theoretical distance between the upper and lower electrodes should be 2h, with h being the thickness of the pouch film. In consideration of the non-ideal capacitor and the impact of the residual dielectric liquid situated between the films, a thickness correction factor, designated as $K_{h}$, is introduced. It is observed that the relative dielectric constant of the film is represented as $\varepsilon_{r}$, while the absolute dielectric constant is represented as $\varepsilon_{0}$. Additionally, the applied voltage is U, the equivalent capacitance is C, and the electrostatic force generated in the suction critical region of length dx is equal to the pressure generated by the dielectric liquid.

$$\frac{1}{2}\frac{\varepsilon_{0}\varepsilon_{r}\mathrm{dx}}{2hK_{h}}\frac{U^{2}}{2hK_{h}}=\mathrm{pdx}$$ (6)

The force–electric–hydraulic coupling relationship of the electrohydraulic actuator is obtained by solving Eqs. (1) to (6), which allows us to determine the variation in the actuator bending angle with changes in voltage. It is worth mentioning that an uninterrupted increase in voltage does not result in an infinite expansion of the bending angle. As the dielectric liquid fills the space between the electrodes, the upper part of the pouch forms an arc, while the lower part forms 2 radius edges with a length of $R_{f}$. This geometry constrains the maximum bending angle of the actuator to $\varphi =\pi -\frac{2R_{f}}{R_{f}}=\left(\pi -2\right)\;\mathrm{rad}\approx 65{}^{\circ}$.

We tested the variation of the bending angle of the actuator with voltage, as illustrated in Fig. 2E. The results demonstrate that the theory successfully predicts the bending angle of the actuator. In the event that the input voltage is below 3 kV, the bending angle of the actuator is relatively minor. At this point, the theoretical value is larger than the experimental one, because the wedge shape of the actuator and the zip-like absorption process determine that the actuator needs a start-up voltage to be driven. If the driving voltage is lower than the start-up voltage, the electrodes at both sides of the actuator cannot be absorbed, and therefore, the actuator cannot bend effectively. Besides, it is evident that the start-up voltage of this actuator is around 3 kV by experiments. Conversely, when the voltage surpasses 3 kV, the bending angle increases exponentially in conjunction with the rising voltage. Upon reaching 6 kV, the electrodes have been fully attracted, and all the dielectric liquid has entered the area of the uncovered electrodes. This causes the actuator to bend to its maximum angle. At this point, the bending angle of the actuator is no longer markedly increased by further voltage increases. It can be observed that at these voltages the theoretical values are slightly larger than the experimental ones. This is because the theoretical analysis assumes the liquid concentrated at the joint as an ideal arch column, whereas in reality the pressure of the liquid causes wrinkles at the side peripheries of the pouch, which means that the amount of liquid forming the column is less than ideal, resulting in a smaller bending angle of the actuator. It should also be noted that when the voltage exceeds 6 kV, the actuator is susceptible to breakdown failure. To ensure optimal driving performance and stability, subsequent experiments and demonstrations have been conducted with a maximum voltage of 6 kV.

### Input signal for the actuator

During the experiments, we find that the actuators cannot return to its original shape with any great rapidity following the cessation of the power supply. Furthermore, the electrodes on both sides are gradually separated before the actuator is ultimately compelled to expand. This phenomenon can be attributed to the polarization of the pouch film, composed of PET, which is induced by the high voltage. The release of the charge from the film occurs over time, resulting in a prolonged cycle for the actuator and, consequently, an inability to achieve a high-frequency actuation. To address this issue, we consider employing an alternating positive and negative signal configuration for the actuator. As illustrated in Fig. S4, an H-bridge circuit is constructed utilizing 4 high-voltage (HV) relays, with a time-delay relay employed to regulate the activation and deactivation of the corresponding HV relay. When all 4 HV relays are in a deactivated state, the circuit outputs a zero voltage. Conversely, when the 2-diagonal HV relays are activated, the circuit outputs either a positive or negative voltage signal. We program the 4 time-delay relays to set the output sequence in the form of alternating positive-zero-negative-zero, with the positive and negative voltages acting for equal periods of time during an actuating cycle, in order to weaken the polarization effect of the film. It is crucial to highlight that a zero voltage between the positive and negative signals is essential to provide the actuator with sufficient time to flatten itself. In the absence of the zero voltage, the actuator will inevitably bend once more before it is recovered, resulting in a permanent deformation.

### Performance of the actuator

The output torque of the actuator is evaluated through the utilization of the designed signals. As illustrated in Fig. 2F, the back of the skeleton is fixed to a flat surface, while the front end is in contact with the force sensor. The distance between the articulation point and the contact point of the force sensor is 35 mm. To prevent the loss of output force due to the deformation of the flipper membrane, a 1-mm-thick PET sheet is affixed to the front skeleton, with the force sensor pressing against the front end of this sheet. Excitation of the actuator results in transmission of force to the force sensor, enabling calculation of the change in moment considering the distance between the force point and the pivot point. As illustrated in Fig. 2G, the duration of the positive signal, negative signal, and zero level time is identical, each lasting 0.5 s. During one actuation cycle, the moment peaks at the stage of applying positive and negative voltages, and the maximum moment output is in excess of 10 N·mm, which is sufficient to support the crawling robot. Furthermore, after a prolonged operational period (approximately 500 cycles, as seen in Fig. S11), the output moment of the actuator demonstrates minimal deterioration. The actuator exhibits robust actuation capabilities, substantiating its suitability as a flipper of the crawling robot.

## Results and Discussion

### Omnidirectional crawling on the ground

**Fig. 3.**
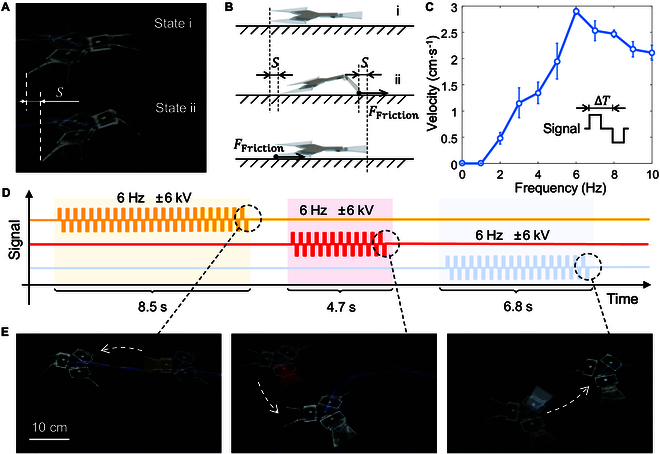
The crawling motion of the amphibious robot on land. (A) Two stages of the robot crawling on land. (B) Schematic diagram of the robot’s crawling principle on land. (C) Measured velocity of the crawling robot, considering the motion frequency. (D) Input signal for the omnidirectional motion of the crawling robot. (E) Omnidirectional motion of the crawling robot, with the motion frequency of 6 Hz.

The fundamental function of the designed robot is to crawl on the ground. This is achieved by the unbalanced friction between the front and rear flippers at different stages, which drives the robot forward. As illustrated in Fig. 3A, the single flipper-actuated robot is subdivided into 2 stages during its locomotive cycle. During stage i, the robot’s flipper undergoes a relaxation process and returns to its original state. Subsequently, during stage ii, one of the robot’s flippers is actuated and reaches a bent state. The specific working principle is shown in Fig. 3B. Upon transitioning from state i to state ii, the tip of the front flipper contacts the ground, resulting in a markedly larger friction force between the front flipper and the ground compared to that between the back flipper and the ground. This is due to the unique skeleton tip design, which enables the front flipper to exert a forward pulling force on the entire robot by a distance S. When the robot is restored to state i from stage ii, the angle between the back flipper and the ground is minimal, thereby reducing the friction between the back flipper and the ground to a level that is insufficient to impede the forward movement of the robot. Conversely, the recovery of the front flipper is in fact upward, rather than forward, resulting in no friction between the front flipper and the ground until the flipper falls to the ground due to gravity. Consequently, within this cycle, the robot progresses a total distance of S. The magnitude of this distance is the difference in body length of the robot between the 2 stages.

The analysis of the motion process reveals that the size of the motion period and the variation value of the robot’s body length exert a important influence on the crawling speed. It can be concluded that the motion frequency is the key factor. The impact of varying motion frequencies on the crawling speed of the robot is investigated, as illustrated in Fig. 3C. It should be mentioned that the actuation frequency of the robot differs from the motion frequency. This is because the electrohydraulic actuator bends under both positive and negative voltage, resulting in an actuation signal period of 2ΔT when the motion period is ΔT. Consequently, the motion frequency is twice the actuation frequency. The frequency employed in this experiment and subsequent demonstrations is that of the motion frequency. Fig. 3C shows that an increase in frequency results in an initial rise in the crawling speed of the robot, followed by a subsequent decline. As the frequency increases from 0 to 6 Hz, the motion speed gradually increases. This is due to the fact that the robot completes an increased number of motion cycles in the same period, resulting in a longer forward distance. As the driving frequency increases to 6 Hz, the moving speed reaches a peak of 2.9 cm/s. It is important to mark that when the motion frequency is 1 Hz, the robot’s speed is 0 cm/s. This is because the robot remains at each stage for an extended period of time. When transitioning from stage ii to stage i, the front flipper makes contact with the ground before the robot recovers, resulting in a cancellation of the friction generated by the back flipper. This causes the robot to remain stationary. Upon further increasing the frequency of motion to 10 Hz, a decline in the crawling speed of the robot is observed. This can be attributed to the fact that the actuation cycles are sufficiently brief for the actuator to deform fully, resulting in the robot advancing a limited distance in each cycle. Consequently, despite the completion of a greater number of cycles per unit of time, the overall distance traveled decreases.

The optimal frequency has been determined to be 6 Hz, as evidenced by experimental results. An actuation signal of 6 kV is employed to propel the robot in a crawling motion on the ground. Fig. 3D illustrates the variation of the input signals, with 3 signals controlling each of the 3 flippers of the robot. As demonstrated in Movie S1, the robot is capable of moving in 3 directions: left, right-down, and right-up. These 3 movements form a set of basis vectors, indicating that the robot is able to reach any point on the plane.

Besides, double-flipper actuation is also proven to move the robot. We have experimented 2 flippers actuated simultaneously at 6 Hz and found that the robot moved in the direction of these flippers and measured a crawling speed of 1.27 cm/s, compared to 2.90 cm/s for a robot actuated by a single flipper at the same frequency. The double-flipper actuation is less efficient than single-flipper actuation because of the geometry structure of the robot, which is explained in detail in the Supplementary Materials, and we choose the single-flipper actuation as the ideal moving method on ground.

### Crawling underwater

**Fig. 4.**
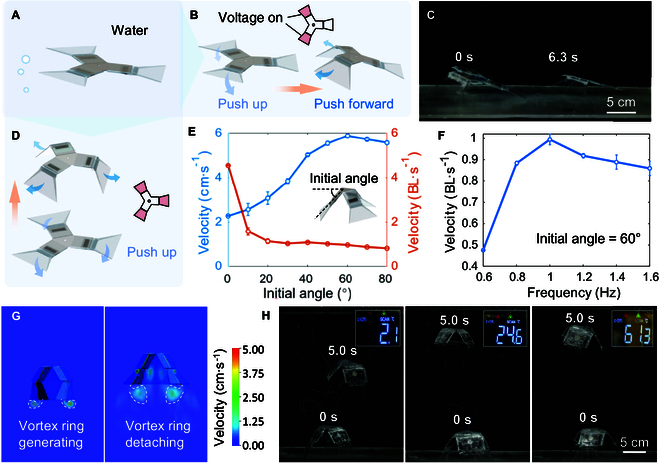
The crawling and swimming motion of the amphibious robot in water. (A) Schematic diagram of the unpowered robot in water. (B) Schematic diagram of the robot’s crawling principle underwater, with 2 flippers actuated. (C) Process of the robot crawling underwater. (D) Schematic diagram of the robot’s swimming principle in water, with all 3 flippers actuated. (E) Measured velocity of the swimming robot, considering the initial angle of the body skeleton. (F) Measured velocity of the swimming robot with initial angle of 60°, considering the motion frequency. (G) Simulation of the swimming robot. (H) Process of the robot swimming in water with temperature of 2.1, 24.6, and 61.3 °C, respectively.

The robot is capable of not only crawling on the ground but also moving in water. As shown in Fig. 4A, the number of actuated flippers determines the type of movement that can be executed. To illustrate, when operated by 2 flippers, the robot is capable of crawling underwater. Nevertheless, the principle underlying the robot’s underwater crawling differs from that of its land crawling. The robot is subject to the effects of buoyancy, experiencing reduced friction with the underwater surfaces. Consequently, the primary source of propulsion in the water is the force generated between the robot’s flippers and the surrounding water. As shown in Fig. 4B, the crawling of the robot in water is divided into 2 stages. During the first stage, when the flippers are driven and bent downward, the force of the water on the flippers is upward, causing the rear end of the robot to float up and move away from the contact surface. Upon restoration of the flippers to their original shape, the flippers swing in a backward direction. The force of the water on the flippers is in a forward direction, thus propelling the robot forward. Concurrently, the rear end of the robot sinks down due to the gravity, ultimately contacting with the ground, completing a cycle.

Similarly, the frequency of motion during underwater crawling should not be excessive, as this may result in insufficient deformation of the actuator, thereby limiting the angle of the flippers with the ground and the subsequent forward propulsion. The robot is driven to crawl underwater with a motion frequency of 1.6 Hz and a voltage of 6 kV. As shown in Fig. 4C, the time required for the robot to advance 20 cm is 6.3 s, with a speed of 3.2 cm/s.

### Swimming in water

Once all 3 flippers are in place, the robot transitions from crawling to swimming mode, as illustrated in Fig. 4D. This is achieved by the simultaneous fluttering of all 3 flippers, which generates a symmetrical upward propulsive force, causing the robot to move upward. When power is stopped, the dielectric liquid inside the actuator slowly fills back in and the flippers return smoothly to their original state. Given that the flippers will generate a reverse propulsion force on the robot when they return to their original state, we conducted further research into the mechanism of the robot’s actions in the water. It is evident that modifying the initial angle of the actuator can alter the direction of the force exerted on the flippers during the bending and recovery stages, and that different initial angles will consequently affect the swimming speed of the robot in an aqueous environment. The impact of initial angles, spanning a range from 0° to 80°, on the robot’s swimming speed is evaluated. It is established that angles exceeding 80° will result in interference between the flippers during bending, which is deemed to be an unfeasible range. The findings are illustrated in Fig. 4E. When the initial angle is 0°, corresponding to the robot being fully spread, its absolute speed is 2.3 cm/s. Given that the robot’s height is minimal in this configuration, its speed relative to its own body height reaches 4.6 body length per second (BL/s). The fastest absolute velocity of the robot is observed when the initial angle reached 60°, at 5.9 cm/s, which surpasses the majority of electrostatically driven underwater thrusters. Subsequently, the impact of motion frequency on the robot’s swimming speed is investigated, utilizing an actuating voltage of 6 kV and an initial angle of 60°. As shown in Fig. 4F, a reduction in actuating frequency results in a longer interval between each actuation, leading to a deceleration due to gravitational acceleration. Conversely, an increase in actuating frequency causes a decrease in the amplitude of the flipper flutter, accompanied by a reduction in propulsion efficiency. The experimental results demonstrates that the optimal actuating frequency for the robot is 1 Hz, resulting in the fastest swimming speed of 0.99 BL/s.

Actually, the swimming mechanism of the robot in water is much more complex. A fluid simulation of the swimming process of the robot is conducted, and the vortex intensity cross-section of the flow field during one motion cycle is illustrated in Fig. 4G. In the initial phase of the cycle, the robot’s flippers assume a downward bend, and in the subsequent phase, the flippers relax and expand, accompanied by the generation and recapture of a vortex ring beneath the robot. The initial vortex ring is generated at the edge of the flippers during the downward fluttering process of the robot. During the recovery process, the initial vortex ring detaches from the front of the flippers and converges to the middle. The tendency of water to be filled into the chamber beneath the robot, driven by the fluid, is accompanied by the recapture and utilization of the energy of the fluid, thereby enhancing the robot’s swimming efficiency. Furthermore, the dislodging and interaction of the vortex rings facilitate the generation of thrust for the robot’s upward movement.

Furthermore, the kinematic performance of the robot is evaluated at varying ambient temperatures, as shown in Fig. 4H. The robot is observed to swim in water at temperatures of 2.1, 24.6, and 61.3 °C, with a motion frequency of 1 Hz and an actuation voltage of 6 kV, respectively. At elevated temperatures, the robot’s kinematic performance remains comparable to that observed at room temperature. However, at lower temperatures, the swimming speed of the robot declines markedly. This is attributed to the fact that the viscous coefficient of the dielectric liquid within the pouch increases at lower temperatures, resulting in the electrodes being unable to flow the liquid with the same efficiency when squeezing. This leads to a reduction in the transient performance of the actuator. Nevertheless, the robot is still capable of attaining a swimming speed of 2.7 cm/s at 2.1 °C, which markedly demonstrates the great environmental adaptability of the proposed robot.

### Motion mode transition

As previously stated, the designed robot is capable of crawling on land and in water, or swimming in water. The robot’s ability to switch between these modes of motion does not require structural alterations or changes to the type of actuator; instead, it merely necessitates the switching of control strategies. This enables the robot to move continuously on land and in water. We demonstrate the robot’s ability to crawl from land to water, and transition from crawling to swimming in water.

Movie S2 illustrates the transition of the robot from a terrestrial to an aquatic environment. On land, the robot moves at a frequency of 1 Hz and crawls down a slope. Upon reaching the transition area between land and water, the robot is supported by its back flippers and pushed down the slope until it is completely submerged in water. In this process, the actuator must overcome the surface tension of the water as it bends, resulting in a decrease of the crawling speed. As the robot is completely submerged in water, the ground polar of the circuit is exposed to the water, and the motion frequency is increased from 1 to 1.6 Hz. It is not necessary to unplug the ground cable, because it will attract the same number of polar particles in principle whether it is connected to the unsealed electrode or not. Hence, when transiting between mediums, controlling the voltage and frequency remotely is the only operation. The robot is propelled forward by the force generated between its flippers and the surrounding water, facilitating its transition from land to water.

In the context of aquatic locomotion, the ground polar of the actuators is invariably coupled with the water. As shown in Movie S3, the robot initially traverses the aquatic environment by relying on its 2 back flippers, which are driven at a frequency of 1.6 Hz. Subsequently, all 3 flippers are engaged, enabling the robot to commence an upward swimming motion. It is evident that the robot’s initial angle of 0° does not facilitate a rapid upward swimming motion. Nevertheless, this demonstration substantiates the viability and potential of our proposed transformation of the robot’s motion modes in water. The utilization of these motion mode shifts enables the robot to adapt to diverse terrains, thereby enhancing its capacity to perform intricate tasks.

## Conclusion

This study presents a robot that has been modified to become amphibious by altering its locomotion pattern. By optimizing the design of the structure and actuators, the robot is equipped with excellent adaptability on land and in water, allowing it to not only crawl omnidirectionally on land but also crawl underwater or swim in water. These shifts in movement modes necessitate only the adjustment of control strategies, rather than alterations to the robot’s structural composition. This enables the robot to transition between movement modes seamlessly, thereby offering considerable potential for multiterrain exploration.

In order to identify the optimal actuation performance, we have developed a model of the bending electrohydraulic actuator that considers the force–electric–hydraulic coupling. This model has enabled us to predict the relationship between the bending angle and the input voltage, which has allowed us to determine the optimal actuating voltage of 6 kV. This voltage can guarantee both high efficiency and stability, and provides guidance for the application of the same type of actuators in other aspects. To address the issue of charge retention in this actuator, an H-bridge circuit capable of outputting positive, negative, or zero voltages has been constructed to mitigate film polarization. Furthermore, torque experiments have demonstrated that the actuator’s performance remains largely unaffected over an extended period. Furthermore, the overall performance of the robot has been optimized, particularly in relation to the impact of the actuation frequency on the crawling speed of the robot. In the corresponding experiments and demonstrations, the different crawling principles of the robot on land and underwater are explained, and the maximum crawling speed of the robot is found as 2.9 cm/s under land conditions. The initial angle of the robot body skeleton and the motion frequency are optimized for the robot’s swimming mode in the water. Through fluid simulation, the process of vortex ring generation and disengagement is revealed, which demonstrates the robot’s complex swimming mechanism. Furthermore, the robot displays remarkable adaptability to temperature fluctuations. In the existing experiments, it has demonstrated the capacity to withstand temperatures ranging from 2.1 to 61.3 °C, thereby expanding the scope of its potential applications.

Although the designed robot displays considerable promise, there is still considerable scope for further improvement. For instance, the output moment of the actuator shows the phenomenon of alternating large and lower peaks, indicating that the polarization of the film is not completely eliminated. Therefore, the input signal for the actuator needs to be further optimized in the future, and materials with higher dielectric constants may be utilized for the pouch film. Also, high dielectric material and thinner pouch film can lead to a lower driving voltage, which reduces the driving conditions. Furthermore, it is observed that when multiple actuators are connected in parallel, they are more susceptible to failure than a single actuator. In the future, the underlying cause of this phenomenon will require further investigation. Additionally, optimization of materials and processes will be undertaken to enhance the stability and longevity of the robots, ensuring their readiness for exploration in the actual natural environment.

## Data Availability

All data needed to evaluate the conclusions in the paper are present in the paper and/or the Supplementary Materials. Additional data related to this paper may be requested from authors.

## Supplementary Materials

Supplementary 1 Texts S1 to S6 Figs. S1 to S13 Tables S1 to S3 Movies S1 to S3

## References

[1] Li G, Wong T-W, Shih B, Guo C, Wang L, Liu J, Wang T, Liu X, Yan J, Wu B, et al. Bioinspired soft robots for deep-sea exploration. Nat Commun. 2023;14(1):7097. 37925504 10.1038/s41467-023-42882-3 PMC10625581

[2] Del Dottore E, Mondini A, Rowe N, Mazzolai B. A growing soft robot with climbing plant-inspired adaptive behaviors for navigation in unstructured environments. Sci Robot. 2024;9(86): Article eadi5908. 38232147 10.1126/scirobotics.adi5908

[3] Jung Y, Kwon K, Lee J, Ko SH. Untethered soft actuators for soft standalone robotics. Nat Commun. 2024;15(1):3510. 38664373 10.1038/s41467-024-47639-0 PMC11045848

[4] Liu R, Xiang Y, Wei Z, Zhang J. A computer-aided teleoperation system for intuitively controlling the behavior of a magnetic millirobot within a stomach phantom. Adv Intell Syst. 2023;6(2):2300325.

[5] Jeong M, Tan X, Fischer F, Qiu T. A convoy of magnetic millirobots transports endoscopic instruments for minimally-invasive surgery. Adv Sci. 2024;11(35):2308382. 10.1002/advs.202308382 PMC12462913 38946679

[6] Yang Z, Xu C, Lee JX, Lum GZ. Magnetic miniature soft robot with reprogrammable drug-dispensing functionalities: Toward advanced targeted combination therapy. Adv Mater. 2024; Article e2408750. 39246210 10.1002/adma.202408750

[7] Xu H, Zhi J, Chen B, Zhao S, Huang J, Bi C, Li L, Tian B, Liu Y, Zhang Y, et al. A biomimetic adhesive disc for robotic adhesion sliding inspired by the net-winged midge larva. Soft Robot. 2025;12(1):95–108. 39411787 10.1089/soro.2023.0253

[8] Ai X, Yue H, Wang WD. Crawling soft robot exploiting wheel-legs and multimodal locomotion for high terrestrial maneuverability. IEEE Trans Robot. 2023;39(6):4230–4239.

[9] Hawkes EW, Blumenschein LH, Greer JD, Okamura AM. A soft robot that navigates its environment through growth. Sci Robot. 2017;2(8): Article eaan3028. 33157883 10.1126/scirobotics.aan3028

[10] Gong S, Fang F, Yi Z, Feng B, Li A, Li W, Shao L, Zhang W. An intelligent spinal soft robot with self-sensing adaptability. The Innovation. 2024;5(4): Article 100640. 38881800 10.1016/j.xinn.2024.100640 PMC11180339

[11] Li Q, Zhang F, Jing Z, Yu F, Chen Y. A hybrid territorial aquatic bionic soft robot with controllable transition capability. J Bio Eng. 2023;20(2):568–583.

[12] Baines R, Patiballa SK, Booth J, Ramirez L, Sipple T, Garcia A, Fish F, Kramer-Bottiglio R. Multi-environment robotic transitions through adaptive morphogenesis. Nature. 2022;610(7931):283–289. 36224418 10.1038/s41586-022-05188-w

[13] Qu J, Xu Y, Li Z, Yu Z, Mao B, Wang Y, Wang Z, Fan Q, Qian X, Zhang M, et al. Recent advances on underwater soft robots. Adv Intell Syst. 2024;6(2):2300299.

[14] Wu M, Xu X, Zhao Q, Afridi WH, Hou N, Afridi RH, Zheng X, Wang C, Xie G. A fully 3D-printed tortoise-inspired soft robot with terrains-adaptive and amphibious landing capabilities. Adv Mater Technol. 2022;7(12):2200536.

[15] Guo X, Li W, Fang F, Chen H, Zhao L, Fang X, Yi Z, Shao L, Meng G, Zhang W. Encoded sewing soft textile robots. Sci Adv. 2024;10(1): Article eadk3855. 38181076 10.1126/sciadv.adk3855 PMC10776007

[16] Fang F, Li W, Guo X, Chen H, Meng G, Zhang W. Hierarchically reconfigurable soft robots with reprogrammable multimodal actuation. Adv Funct Mater. 2025;35(4):2414279.

[17] Kim M, Yu A, Kim D, Nelson BJ, Ahn SH. Multi-agent control of laser-guided shape-memory alloy microrobots. Adv Funct Mater. 2023;33(51):2304937.

[18] Xu Y, Zhuo J, Fan M, Li X, Cao X, Ruan D, Cao H, Zhou F, Wong TW, Li T. A bioinspired shape memory alloy based soft robotic system for deep-sea exploration. Adv Intell Syst. 2024;6(5):2300699.

[19] Tang D, Zhang C, Pan C, Hu H, Sun H, Dai H, Fu J, Majidi C, Zhao P. Bistable soft jumper capable of fast response and high takeoff velocity. Sci Robot. 2024;9(93): Article eadm8484. 39167670 10.1126/scirobotics.adm8484

[20] Ke X, Yong H, Xu F, Chai Z, Jiang J, Ni X, Wu Z. Synergistical mechanical design and function integration for insect-scale on-demand configurable multifunctional soft magnetic robots. Soft Robot. 2024;11(1):43–56. 37418155 10.1089/soro.2022.0105

[21] Wang Y, Xuan H, Zhang L, Huang H, Neisiany RE, Zhang H, Gu S, Guan Q, You Z. 4D printed non-euclidean-plate jellyfish inspired soft robot in diverse organic solvents. Adv Mater. 2024;36(16): Article e2313761. 38211632 10.1002/adma.202313761

[22] Zhang C, Wang J, Zhao L, Chen R, Zhao Q, Zhao J, Liu F, Bian Y, Zhang H, Wang Y, et al. All-optical driven soft crawler with complex motion capabilities. Int J Smart Nano Mater. 2024;15(2):348–364.

[23] Ren L, He Y, Wang B, Xu J, Wu Q, Wang Z, Li W, Ren L, Zhou X, Liu Q, et al. 4D printed self-sustained soft crawling machines fueled by constant thermal field. Adv Funct Mater. 2024;34(33):2400161.

[24] Park G, Park H, Seo J, Yang JC, Kim M, Lee BJ, Park S. Bidirectional thermo-regulating hydrogel composite for autonomic thermal homeostasis. Nat Commun. 2023;14(1):3049. 37236988 10.1038/s41467-023-38779-w PMC10220006

[25] Xia Y, Mu T, Liu Y, Leng J. Carbon fiber reinforced liquid crystalline elastomer composites: A dual exploration in strength augmentation and transformation flexibility through 4D printing. Int J Smart Nano Mater. 2024;15(2):312–329.

[26] Yang X, Lan L, Pan X, di Q, Liu X, Li L, Naumov P, Zhang H. Bioinspired soft robots based on organic polymer-crystal hybrid materials with response to temperature and humidity. Nat Commun. 2023;14(1):2287. 37085510 10.1038/s41467-023-37964-1 PMC10121608

[27] Zhang Y, Zhou X, Liu L, Wang S, Zhang Y, Wu M, Lu Z, Ming Z, Tao J, Xiong J. Highly-aligned all-fiber actuator with asymmetric photothermal-humidity response and autonomous perceptivity. Adv Mater. 2024;36(33): Article e2404696. 38923035 10.1002/adma.202404696

[28] Yang S, Yang Y, Xia X, Zou B, Wang B, Zhang Y. Biomimetic stimulus responsiveness: From materials design to device integration. Adv Funct Mater. 2024;34(33):2400500.

[29] Guo Q, Sun Y, Zhang T, Xie S, Chen X, Zhang Z, Jiang H, Yang L. Bistable insect-scale jumpers with tunable energy barriers for multimodal locomotion. Adv Sci. 2024;11(34):2404404. 10.1002/advs.202404404 PMC11425846 38973215

[30] Li WB, Guo XY, Zhang WM. SomBot: A bio-inspired dynamic somersaulting soft robot. IEEE Robot Autom Lett. 2021;6(2):1654–1661.

[31] Wu J, Wu M, Chen W, Wang C, Xie G. Multimodal soft amphibious robots using simple plastic-sheet-reinforced thin pneumatic actuators. IEEE Trans Robot. 2024;40:1874–1889.

[32] Chen T, Yang X, Zhang B, Li J, Pan J, Wang Y. Scale-inspired programmable robotic structures with concurrent shape morphing and stiffness variation. Sci Robot. 2024;9(92): Article eadl0307. 39018371 10.1126/scirobotics.adl0307

[33] Sun J, Lerner E, Tighe B, Middlemist C, Zhao J. Embedded shape morphing for morphologically adaptive robots. Nat Commun. 2023;14(1):6023. 37758737 10.1038/s41467-023-41708-6 PMC10533550

[34] Xiao B, Xu Y, Edwards S, Balakumar L, Dong X. Sensing mucus physiological property in situ by wireless millimeter-scale soft robots. Adv Funct Mater. 2023;34(8):2307751. 39990597 10.1002/adfm.202307751 PMC11845219

[35] Wang C, Mzyk A, Schirhagl R, Misra S, Venkiteswaran VK. Biocompatible film-coating of magnetic soft robots for mucoadhesive locomotion. Adv Mater Technol. 2023;8(12):2201813.

[36] Soon RH, Yin Z, Dogan MA, Dogan NO, Tiryaki ME, Karacakol AC, Aydin A, Esmaeili-Dokht P, Sitti M. Pangolin-inspired untethered magnetic robot for on-demand biomedical heating applications. Nat Commun. 2023;14(1):3320. 37339969 10.1038/s41467-023-38689-x PMC10282021

[37] Peng Z, Zhang H, Wang M, Zhang S, Jiang Y, Li Y, Zhu X, Zhang G, Niu G, Zhang J, et al. 3D printing bio-inspired micro soft robot with programming magnetic elastic composites. Adv Mater Technol. 2024;9(9):2301777.

[38] Ren Z, Ucak K, Yan Y, Sitti M. Undulatory propulsion at milliscale on water surface. Adv Sci. 2024;11(19): Article e2309807. 10.1002/advs.202309807 PMC11109636 38483259

[39] Gu GY, Zhu J, Zhu LM, Zhu XY. A survey on dielectric elastomer actuators for soft robots. Bioinspir Biomim. 2017;12(1): Article 011003. 28114111 10.1088/1748-3190/12/1/011003

[40] Tang C, Du B, Jiang S, Shao Q, Dong X, Liu X-J, Zhao H. A pipeline inspection robot for navigating tubular environments in the sub-centimeter scale. Sci Robot. 7(66): Article eabm8597. 35613300 10.1126/scirobotics.abm8597

[41] Wang D, Zhao B, Li X, Dong L, Zhang M, Zou J, Gu G. Dexterous electrical-driven soft robots with reconfigurable chiral-lattice foot design. Nat Commun. 2023;14(1):5067. 37604806 10.1038/s41467-023-40626-x PMC10442442

[42] Wang Y, Zhang P, Huang H, Zhu J. Bio-inspired transparent soft jellyfish robot. Soft Robot. 2022;10(3):590–600. 36577053 10.1089/soro.2022.0027

[43] Li X, Rao D, Zhang M, Xue Y, Cao X, Yin S, Wong JW, Zhou F, Wong TW, Yang X, et al. A jelly-like artificial muscle for an untethered underwater robot. Cell Rep Phys Sci. 2024;5(5): Article 101957.

[44] Li G, Chen X, Zhou F, Liang Y, Xiao Y, Cao X, Zhang Z, Zhang M, Wu B, Yin S, et al. Self-powered soft robot in the Mariana Trench. Nature. 2021;591(7848):66–71. 33658693 10.1038/s41586-020-03153-z

[45] Cheng Z, Feng W, Zhang Y, Sun L, Liu Y, Chen L, Wang C. A highly robust amphibious soft robot with imperceptibility based on a water-stable and self-healing ionic conductor. Adv Mater. 2023;35(28): Article e2301005. 37027814 10.1002/adma.202301005

[46] Wang X, Li S, Chang J-C, Liu J, Axinte D, Dong X. Multimodal locomotion ultra-thin soft robots for exploration of narrow spaces. Nat Commun. 2024;15(1):6296. 39060231 10.1038/s41467-024-50598-1 PMC11282246

[47] Ma W, Li B, Jiang L, Wu Y, Bai R, Chen G. A soft, centimeter-scaled, thin-cable-crawling robot for narrow space inspection. Adv Intell Syst. 2024;6(6):2300828.

[48] Acome E, Mitchell SK, Morrissey TG, Emmett MB, Benjamin C, King M, Radakovitz M, Keplinger C. Hydraulically amplified self-healing electrostatic actuators with muscle-like performance. Science. 2018;359(6371):61–65. 29302008 10.1126/science.aao6139

[49] Kellaris N, Gopaluni Venkata V, Smith GM, Mitchell SK, Keplinger C. Peano-HASEL actuators: Muscle-mimetic, electrohydraulic transducers that linearly contract on activation. Sci Robot. 2018;3(14): Article eaar3276. 33141696 10.1126/scirobotics.aar3276

[50] Rothemund P, Kellaris N, Mitchell SK, Acome E, Keplinger C. HASEL artificial muscles for a new generation of lifelike robots—Recent progress and future opportunities. Adv Mater. 2021;33(19):2003375. 33166000 10.1002/adma.202003375 PMC11469257

[51] Ye Y, He Q, Yang C, Xu X, Xie L, Liu D, Zeng B, Leng Z. Optimized design and performance testing of hydraulic electrostatic actuator. Int J Smart Nano Mater. 2024;15(4):811–834.

[52] Xiong Q, Zhou X, Li D, Ambrose JW, Yeow RC. An amphibious fully-soft centimeter-scale miniature crawling robot powered by electrohydraulic fluid kinetic energy. Adv Sci. 2024;11(4): Article e2308033. 10.1002/advs.202308033 PMC11005735 38303577

[53] Yoder Z, Rumley EH, Schmidt I, Rothemund P, Keplinger C. Hexagonal electrohydraulic modules for rapidly reconfigurable high-speed robots. Sci Robot. 2024;9(94): Article eadl3546. 39292807 10.1126/scirobotics.adl3546

[54] Chen R, Zhu X, Yuan Z, Pu H, Luo J, Sun Y. A bioinspired single actuator-driven soft robot capable of multistrategy locomotion. IEEE Trans Robot. 2024;40:2149–2165.

[55] Zhang Y, Li W, Liu F, Chen P, Zhu H, Li H, Li G, Tao K, Zhang W, Xu J. A manta ray-inspired fast-swimming soft electrohydraulic robotic Fish. IEEE Robot Autom Lett. 2024;9(9):8043–8050.

[56] Wang T, Joo H-J, Song S, Hu W, Keplinger C, Sitti M. A versatile jellyfish-like robotic platform for effective underwater propulsion and manipulation. Sci Adv. 9(15): Article eadg0292. 37043565 10.1126/sciadv.adg0292 PMC10096580

[57] Gravert S-D, Varini E, Kazemipour A, Michelis MY, Buchner T, Hinchet R, Katzschmann RK. Low-voltage electrohydraulic actuators for untethered robotics. Sci Adv. 2024;10(1):eadi9319. 38181082 10.1126/sciadv.adi9319 PMC10775996

[58] Kellaris N, Rothemund P, Zeng Y, Mitchell SK, Smith GM, Jayaram K, Keplinger C. Spider-inspired electrohydraulic actuators for fast, soft-actuated joints. Adv Sci. 2021;8(14): Article e2100916. 10.1002/advs.202100916 PMC8292915 34050720

