# Supplementary material for: A Multimodal Amphibious Robot Driven by Soft Electrohydraulic Flippers
Source: Cyborg Bionic Syst. 2025 Jun 9;6:0253. doi: 10.34133/cbsystems.0253 (PMC12146520; doi:10.34133/cbsystems.0253)
Supplement: Supplementary 1 — Texts S1 to S6 Figs. S1 to S13 Tables S1 to S3 Movies S1 to S3 [file cbsystems.0253.f1.zip › Supplementary Materials - clean version.pdf]

A Multimodal Amphibious Robot Driven by Soft Electrohydraulic Flippers

Fuyi Fang[1], Junfeng Zhou[1], Yuanzhen Zhang[2], Yanran Yi[1], Zhen Huang[1], Yicheng Feng[1], Kai Tao[3]*, Wenbo Li[2]*, Wenming Zhang[1]*

[1]State Key Laboratory of Mechanical System and Vibration, School of Mechanical Engineering, Shanghai Jiao Tong University, Shanghai 200240, China
[2]School of Aerospace Engineering and Applied Mechanics, Tongji University, Shanghai 200092, China
[3]The Ministry of Education Key Laboratory of Micro and Nano Systems for Aerospace, School of Mechanical Engineering, Northwestern Polytechnical University, Xi'an 710072, China

*Address correspondence to: wenmingz@sjtu.edu.cn (W.Z.); wenboli@tongji.edu.cn (W.L.); taokai@nwpu.edu.cn (K.T.).

Text 1. Manufacturing process and working principle of the flipper

Materials selection: The flipper consists of an electrohydraulic actuator and skeletons. Among them, the electrohydraulic actuator is composed of several layers, including a pouch, electrodes and waterproof layers. A PET-EVA composite film with a thickness of 27 μm is used for the pouch film. We choose PET film because it has lower stretchability compared to PE or TPU materials, which prevents energy loss due to stretching of the material during deformation, while the PET film material also contains excellent flexibility. the EVA coating is to facilitate the sealing between the films. The thickness of the film is based on the work of Kellaris[58] and Zhang[55], which ensures that the actuator is both robust and has sufficient output. The dielectric fluid filled in the pouch is silicone oil with a viscosity of 1 cs, which is a common dielectric liquid used in electrohydraulic actuators, and the lower the viscosity, the better the dynamic performance of the actuator. The electrode material used is carbon paste, which has good electrical conductivity, meanwhile, it is compatible with screen printing processes and is less expensive than materials such as silver paste and PEDOT:PSS. The waterproof layer consists of 60 μm PET tape and waterproof soft adhesive. We have tested 40 μm, 50 μm and 60 μm PET tape and found that 60 μm PET tape guarantees the avoidance of electrical breakdown. The waterproof soft adhesive is a UV-curable adhesive that remains fluid until cured, allowing it to fill gaps and improve sealing.

To ensure that the skeleton has sufficient rigidity and remains lightweight, we use 1mm thick acrylic sheet and 0.1mm thick TPU film. In order to connect the two parts of the skeleton securely while maintaining flexibility, 0.125 mm thick PET tape is used

to connect the joints. The materials for all components are listed in Supplementary Materials Table S2.

Structural design: The sizes of the electrohydraulic actuator refer to the researches from Kellaris[58], Wang[56] and Zhang[55]. The 20 × 40 mm electrodes part and the 10 mm wide hydraulic part allow the actuator a large output force. Although increasing the length and width of the electrode part can further increase the output force of the actuator, an actuator that is too long or too wide will cause interference of the components during robot movement, so the sizes are appropriate for this robot. The amount of dielectric liquid in the pouch should be just enough to fill the hydraulic part, forming an arch-shaped cross-section. The lack of liquid will prevent the hydraulic part from being fully filled, reducing the output force and bending angle of the actuator, while too much liquid will prevent the electrodes from being fully absorbed, again reducing the efficiency of the actuator. In this work, the appropriate liquid volume is 1.2 ml in the actuator size.

The key to the structure of the flipper skeleton is the size of the front part. The design must consider that the length of the front part should not be too short, as this will make it difficult to generate enough torque. However, if the front part is too long, it will make the robot too large and inflexible to move. The area of the flipper needs to be as large as possible to provide more propulsion in the water, but the width of the end of the flipper should not be too large to cause interference of multiple flippers in the bending state. Given these trade-offs, an isosceles trapezoid of 30 (60) × 40 mm is utilized as the front part of the flipper. The sizes of each component are shown in Fig. S1.

Manufacturing Process: The PET sheet and acrylic sheet used in this work are laser cut, and the PET-EVA film and TPU film are cut by hand. The manufacturing process of the flipper is shown in Fig. S2 as follows: ① Stack two layers of PET-EVA film with EVA coating opposite to each other and cover them with PI protective film, then heat-seal the shape of the pouch by an FDM 3D printer; ② Cut the heat-sealed pouch off; ③ Screen print carbon paste electrodes on both sides of the pouch and wait to dry; ④ Inject 1.2 ml of silicone oil into the pouch and heat-seal the oil injection port; ⑤ Fix the cable to the electrodes with conductive tape, then apply waterproof soft adhesive around the electrodes, cover the electrodes with PET tape, and finally apply another layer of waterproof soft adhesive to the side of the PET tape; ⑥ Stick the fabricated electrohydraulic actuator on the skeleton, making sure that the hydraulic part is aligned with the joint of the skeleton.

The entire robot is made of a body skeleton and three flippers, with all the cables across the hole on the center of the body skeleton.

Working principle: The working process of the actuator is similar to that of a “zip”, as shown in Fig. S6. When the electrodes are energized, they begin to absorb from one end and gradually push the dielectric fluid into the front end of the actuator, where the fluid forms a arch-shaped column bounded by the membrane and the skeleton joints, causing the skeleton to bend. The actuator works slightly differently on land and in the water. On land, the electrodes on both sides are connected to cables, so the charge is supplied by the cables. Whereas in water, as one side of the electrode is exposed to the water, the charge on that side is due to polar molecules attracted by the electrode on the encapsulated side, and the exposed cable act as the role to attract the remaining polar molecules to maintain the charge balance in the aqueous environment. Anyway, both principles are effective in causing the flipper to bend.

Text 2. Experiments design and data processing

Experiment design: The experiments in this paper include the flipper bending experiment, the output torque experiment, the temperature resistance experiment, the robot crawling experiment on land, the robot crawling experiment in water and the robot swimming experiment in water. The circuits used are shown in Fig. S4, and the corresponding electronic components are also listed in Table S3.

The experiment platform for flipper bending is shown in Fig. S5A, where the back part of the flipper skeleton was fixed to a flat surface. To test the effect of voltage on the bending angle, the voltage was increased in 1 kV steps until it reached 8 kV, the voltage at which the actuator is susceptible to failure.

The experiment platform of the output moment of the flipper is shown in Fig. S5B. The back part of the flipper skeleton was fixed on a flat surface and the other part was held against the force sensor. The force sensor had a range of 5 N and an accuracy of 1×10^{-6} N, and was connected to a NI signal collector. The sampling frequency of the experiment was 1,000 Hz. The experiment held the actuating frequency of 1 Hz and an input voltage of 6 kV.

The experiment platform for the temperature influence on the flipper is shown in Fig. S5C. The experiment used a test chamber for high/low temperature to hold the flipper in the range of -5°C to 70°C in 5°C steps. And the input voltage was kept as 6 kV.

The platform of the robot crawling experiment on land is shown in Fig. S5D.

During the frequency experiment, the voltage was kept at 6 kV and the crawling speed of the robot was tested at 0~10 Hz actuating frequency. During the voltage experiment, the actuating frequency was kept at 6 Hz and the crawling speed of the robot was tested at 0~6 kV.

The experiment platform for both crawling and swimming of the robot in water is shown in Fig. S5E. In the experiments on the effect of the initial bending angle of the robot skeleton on swimming, the swimming velocity of the robot was tested in the initial angle range of 0~80° in steps of 10°. When conducting experiments on the effect of robot actuating frequency on swimming, the swimming velocity of the robot in the frequency range of 0.6~1.6 Hz was tested in steps of 0.2 Hz by keeping the initial angle of the robot skeleton at 60° and the input voltage at 6 kV. For the experiment on the effect of voltage on the underwater swimming of the robot, the initial angle of the robot skeleton was kept at 60°, the actuating frequency was 1 Hz, and the swimming velocity of the robot was tested in the voltage range of 0~6 kV.

Data processing: Each of the above experiments was performed five times for each group, with a 3-minute interval between the static experiments to ensure that the actuator had released the residual charge completely. Experimental photos and videos were recorded using a *Canon 80D* camera. After calibration, significant deviation data was removed and the average value and standard deviation were calculated by MATLAB.

Text 3. Multi flippers actuation on the ground

Double-flipper actuation moves the robot in the opposite direction to single-flipper actuation. However, due to the limitations of the robot's geometry structure, double-flipper actuation is less efficient than single-flipper actuation. As shown in Fig. S8, when a single flipper is actuated, the total length of the robot in the forward direction is shortened by S, and thus the distance advanced by the robot during a cycle is also S. When two flippers are actuated, the direction of flipper bending is not the same as the direction of robot advance, but is at an angle of $60°$, and the total length of the robot in the forward direction is shortened by $S \cdot cos60° = S/2$, and correspondingly the distance advanced by the robot during a cycle is $S/2$. Therefore, the speed of the robot driven by two flippers is about half that of the robot driven by a single flipper. The experiments shows that double-flipper actuation drives the robot to a speed of 1.27 cm/s with actuation frequency of 6 Hz, while it is 2.90 cm/s to the single-flipper. Some other factors like friction may also affect the speed.

Text 4. The loading performance of the robot

The robot weighs 17.2 g of all, and the distance from the barycenter of the robot to the end of the front flipper is 8.7 cm, and to the end of the back flippers is 7.0 cm, as is seen in Fig. S9A, so the torque required to support the robot is only 12.04 N · mm. The experiment shown in Fig. 2F measured the torque to the fulcrum of the flipper joint, which is 3.5 cm from the force point, so actually the maximum torque of the flipper is over 44.86 N · mm when converting to the fulcrum of the entire robot. Thus, the flippers have excess output torque that can be used to load additional weight. We experimented the crawling speed of the robot with different loads, under the actuating voltage of 6000 V and frequency of 6 Hz. As is seen in Fig. S10, the results show that the robot is still able to move at the speed about 1 cm/s when the load is 15 g, and as the load reaches up to 25 g, the robot barely moves.

Text 5. Influence of the voltage on robot's velocity

The robot's moving velocity varies with the driving voltage. We tested the variation of the crawling velocity and swimming velocity of the robot at driving voltages ranging from 0 to 6 kV. Fig. S12A shows the crawling velocity. When the voltage was low, the robot could not be driven effectively because the flippers bent at a small angle. The robot started to move until the voltage was up to 4 kV, and the velocity increased with the increase of driving voltage. When the voltage was 6 kV, the crawling velocity of the robot reached 2.9 cm/s. Fig. S12B shows the swimming velocity. When swimming upwards, the robot needed to overcome gravity and therefore relied more on the propulsion force of the flippers. When the voltage was low, the flippers were unable to generate sufficient propulsion to counteract the gravitational acceleration applied to the robot, resulting in an upward and then downward movement in a single cycle with an overall velocity of 0 cm/s. The robot did not swim upward until the voltage reached 5 kV, and as the voltage reached 6 kV, the robot's velocity was 5.9 cm/s.

Text 6. Temperature tolerance of the actuator

We tested the temperature tolerance of the actuator. As shown in Fig. S13, the flippers can be markedly actuated in the range of -30~70°C. As the temperature increases, the bending angle of the actuator tends to improve because the viscosity of the dielectric liquid inside the pouch decreases. However, when the temperature

increases to 75°C, the EVA coating on the pouch films begin to melt, causing the dielectric liquid to be squeezed out of the pouch, significantly reducing the performance of the actuator. At low temperature range, the actuator can still work down to -30°C but with the bending angle decline, and it can be expected that the bending angle will continue to decrease as the temperature falls. It is also worth noting that the increased viscosity of the dielectric liquid causes a significant reduction in the dynamic performance of the actuator, resulting in a significant reduction in the efficiency of the robot's motion.

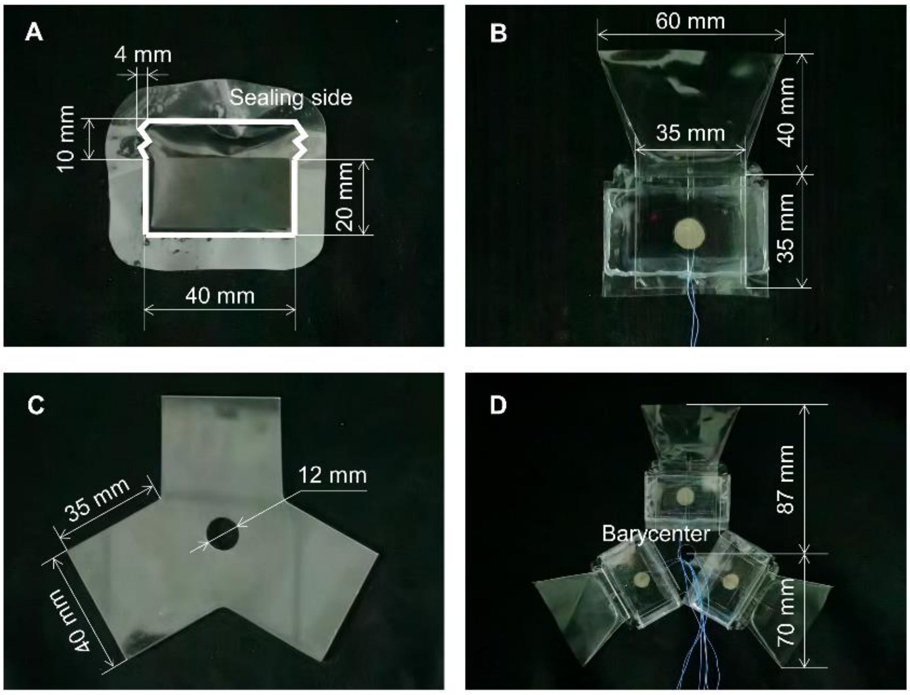

Fig. S1. Sizes of the components. (A) Actuator. (B) Flipper. (C) Skeleton. (D) The entire robot.

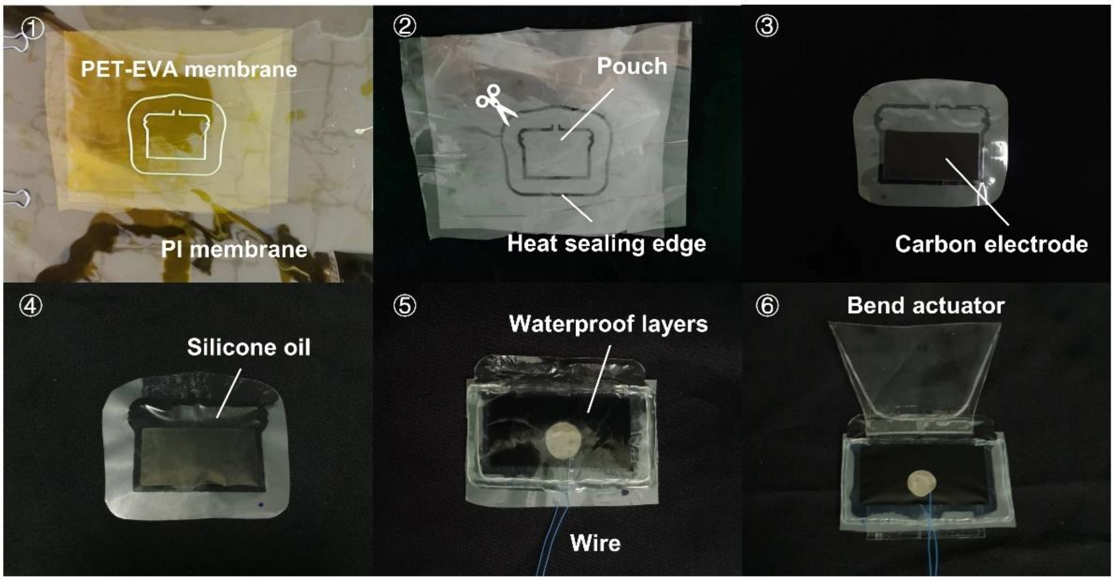

Fig.S2. The manufacturing process of the soft electrohydraulic flippers.

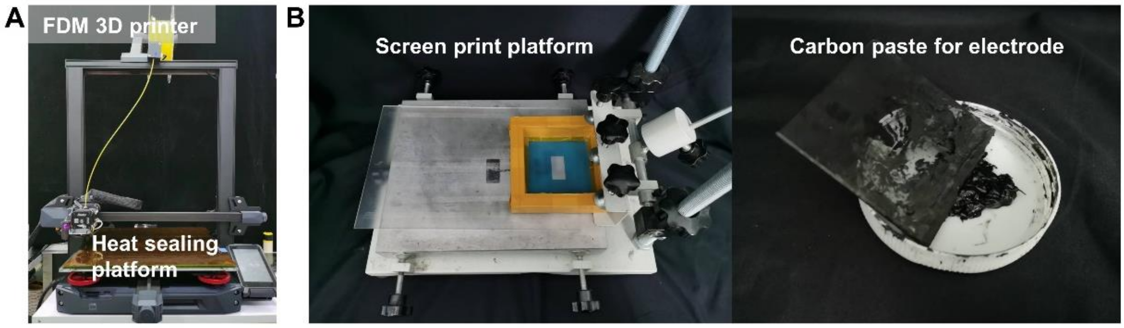

Fig.S3. The manufacturing platform of the electrohydraulic flippers. (A) The FDM 3D printer for heat sealing the pouch. (B) The platform and carbon paste for screen printing the electrodes.

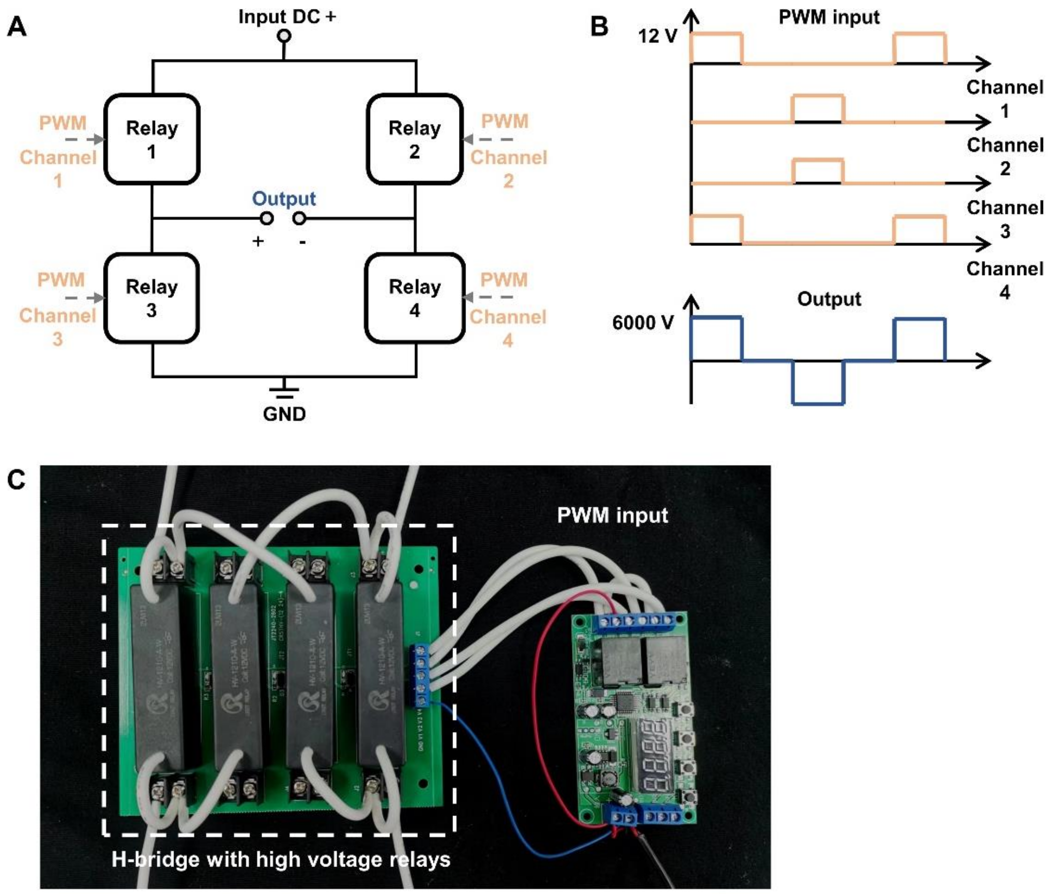

Fig.S4. Circuit for the output positive-zero-negative-zero signal. (A) Scheme of the H-bridge circuit. (B) Input of the four PWM channels and output of the signal. (C) Physical diagram of the circuit.

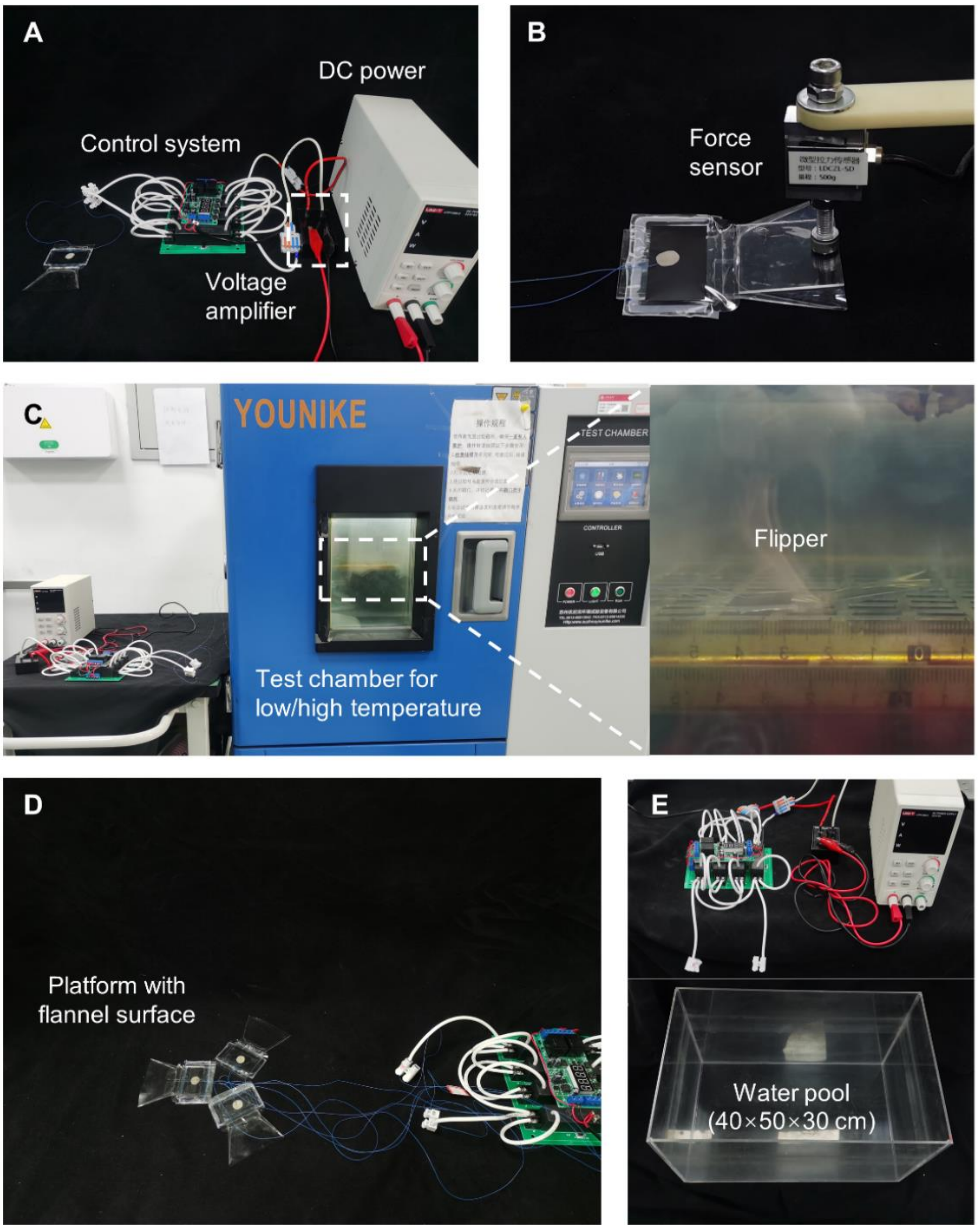

Fig. S5. Experiment platforms (A) For flipper bending (B) For output moment of the flipper. (C) For the temperature influence on the flipper. (D) For the robot crawling experiments on land. (E) For the robot crawling and swimming experiments in water.

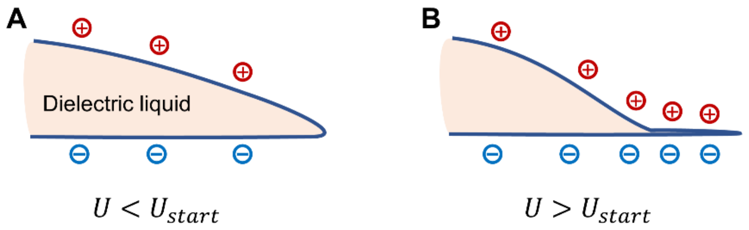

Fig. S6. Absorption process of the actuator. (A) The electrodes fail to absorb as the driving voltage under the start-up voltage. (B) The electrodes start to absorb as the driving voltage exceeds the start-up voltage.

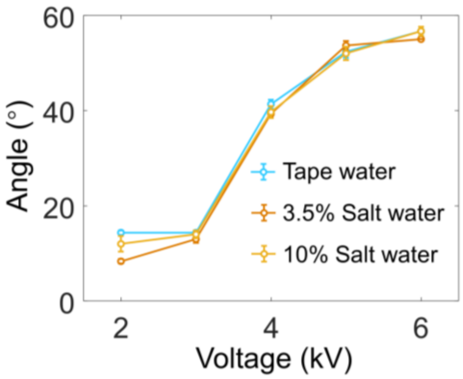

Fig. S7. Variation of the actuator bending angle with voltage in different concentrations of salt water.

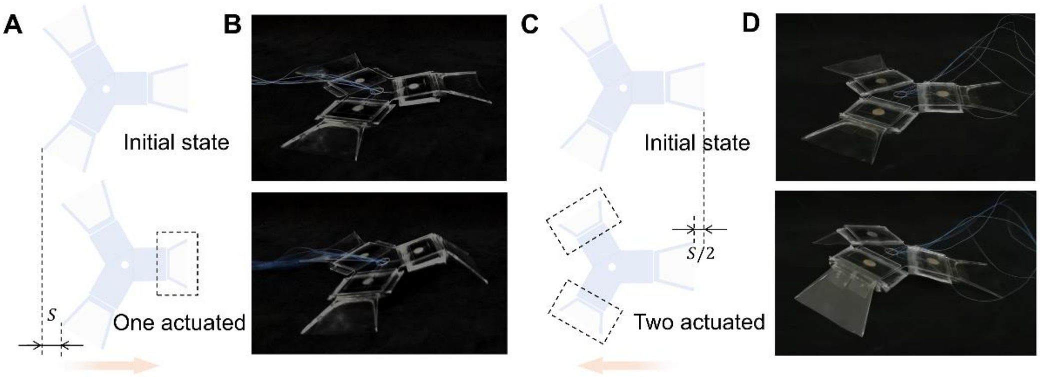

Fig. S8. Crawling process of the single-flipper and double-flipper actuations. (A) Diagram of the single -flipper actuation. (B) Experiment of the single-flipper actuation. (C) Diagram of the double-flipper actuation. (D) Experiment of the double-flipper actuation.

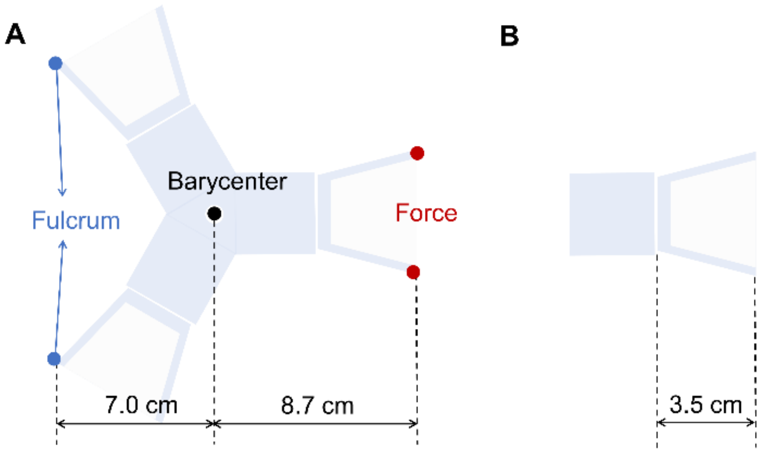

Fig. S9. Schematic diagram of the forces on the crawling robot. (A) Distances between the fulcrum, barycenter, and force point. (B) Distance between the joint and end point of the flipper.

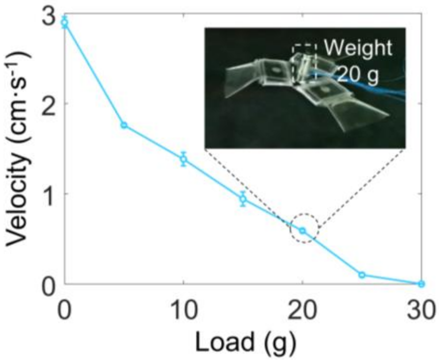

Fig. S10. Variation of robot's velocity with different loads when crawling on land.

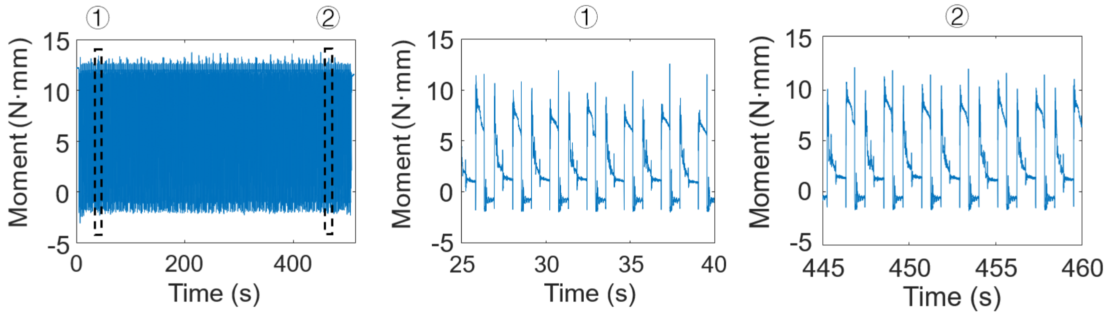

Fig.S11. Repetitive actuation of the soft electrohydraulic flipper for over 500 circles, showing the stable performances.

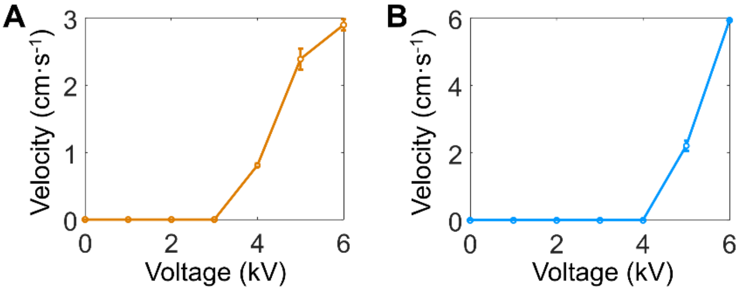

Fig. S12. Variation of robot's moving velocity with voltage. (A) On land. (B) In water.

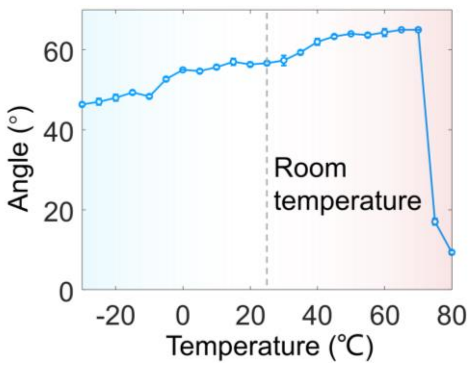

Fig. S13. Temperature tolerance of the actuator

Table S1 Parameters of the soft electrohydraulic flipper model

Symbol	Parameter	Value
l_0	Length of the pouch affixed to the back skeleton	40×10^{-3} m
l_1	Length of the pouch affixed to the front skeleton	5.1×10^{-3} m
l_2	Length of the pouch affixed to the back skeleton and not attracted	-
S_0	Cross-sectional area of the internal dielectric liquid	27×10^{-6} m^2
φ	Angle between two parts of the skeleton	-
θ_1, θ_2	Angle between the pouch arc and the two parts of the skeleton	-
R	Radius of the bulging portion of the pouch	-
γ	Circumcenter angle of the bulging portion of the pouch	-
T_1	Tension inside the film	-
p	Pressure of the dielectric liquid inside the pouch	-
M_1	Equivalent moment of the load	$0.1cos\varphi$ N·m
h	Thickness of the pouch film	27×10^{-6} m
K_h	Thickness correction factor	4.8
ε_r	Relative dielectric constant of the film	3
ε_0	Absolute dielectric constant	8.854×10^{-12} F/m
U	Applied voltage	-
C	Equivalent capacitance	-

Table S2 Materials of the robot

**Component**	**Material**
Pouch film	27 μm (thickness) PET film
Dielectric liquid	1 cs (viscosity) Silicon oil
Electrode	CH-8 carbon paste
Waterproof soft adhesive	Zhuolide D-0084
Waterproof tape	60 μm (thickness) PET film
Flipper skeleton	1 mm (thickness) acrylic sheet
Flipper film	0.1 mm (thickness) TPU film
Adhesive tape	0.125 mm (thickness) PET tape
Robot skeleton	1 mm (thickness) acrylic sheet
Cable	0.25 mm (diameter) Teflon-copper wire

Table S3 Components of the control system

Component	Type/Version
DC power supply	UNI-T UTP1306-II
Voltage amplifier	EMCO F101R
High voltage relay	CRSTHV-20 kV-A
Dual time delay relay	YF-7
